# A small molecule that inhibits the evolution of antibiotic resistance

**DOI:** 10.1093/narmme/ugae001

**Published:** 2024-01-23

**Authors:** Juan Carvajal-Garcia, Harrison Bracey, Anna E Johnson, Angel J Hernandez Viera, Martin Egli, Esra N Simsek, Emily A Jaremba, Kwangho Kim, Houra Merrikh

**Affiliations:** Department of Biochemistry, Vanderbilt University, Nashville, TN 37232, USA; Department of Biochemistry, Vanderbilt University, Nashville, TN 37232, USA; Department of Biochemistry, Vanderbilt University, Nashville, TN 37232, USA; Department of Biochemistry, Vanderbilt University, Nashville, TN 37232, USA; Department of Biochemistry, Vanderbilt University, Nashville, TN 37232, USA; Department of Biochemistry, Vanderbilt University, Nashville, TN 37232, USA; Department of Biochemistry, Vanderbilt University, Nashville, TN 37232, USA; Vanderbilt Institute of Chemical Biology, Vanderbilt University, Nashville, TN 37232, USA; Department of Biochemistry, Vanderbilt University, Nashville, TN 37232, USA

## Abstract

Antibiotic resistance rapidly develops against almost all available therapeutics. Therefore, searching for new antibiotics to overcome the problem of antibiotic resistance alone is insufficient. Given that antibiotic resistance can be driven by mutagenesis, an avenue for preventing it is the inhibition of mutagenic processes. We previously showed that the DNA translocase Mfd is mutagenic and accelerates antibiotic resistance development. Here, we present our discovery of a small molecule that inhibits Mfd-dependent mutagenesis, ARM-1 (anti-resistance molecule 1). We found ARM-1 using a high-throughput, small molecule, *in vivo* screen. Using biochemical assays, we characterized the mechanism by which ARM-1 inhibits Mfd. Critically, we found that ARM-1 reduces mutagenesis and significantly delays antibiotic resistance development across highly divergent bacterial pathogens. These results demonstrate that the mutagenic proteins accelerating evolution can be directly inhibited. Furthermore, our findings suggest that Mfd inhibition, alongside antibiotics, is a potentially effective approach for prevention of antibiotic resistance development during treatment of infections.

## Graphical Abstract

**Graphical Abstract:**
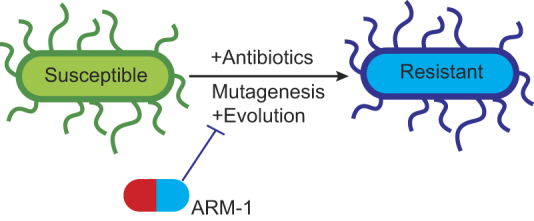

## Introduction

Antibiotic resistance is one of the public health crises of the 21st century. Hundreds of drug-resistant bacterial strains continue to emerge and are currently responsible for 1.3 million deaths every year, with a projection of >10 million deaths from antibiotic-resistant infections by year 2050, costing 210 trillion USD in annual global GDP (1–3). For virtually every antibiotic used in the clinic, resistant strains have been found, which has led to a decrease in the development of new antibiotics, as any new antibiotic that is introduced is predicted to soon become ineffective, and therefore unprofitable (4–6).

One common way bacteria develop resistance to antibiotics is through mutations in their DNA (7). Target and non-target-based mutagenesis (i.e. mutations in genes coding for the antibiotic target or other genes related to resistance acquisition) plays a significant role in resistance development to several antibiotic classes such as aminoglycosides (8), macrolides (9), oxazolidinones (10), β-lactams (11), quinolones (12), tetracycline (9) and chloramphenicol (8) in diverse pathogenic species, such as *Mycobacterium tuberculosis* (13), *Staphylococcus aureus* (11), *Streptococcus pneumoniae* (14), *Pseudomonas aeruginosa* (8) and *Escherichia coli* (7), among others. These mutations leading to antibiotic resistance development can prevent binding of the antibiotic to its target, decreased uptake of antibiotics or modification of enzymatic activities (15).

Our group and others have proposed a fundamental shift in the approach to combating antibiotic resistance: inhibiting evolution by directly inactivating the bacterial mechanisms that increase mutation rates (16–18). This strategy would decrease the chances of acquiring resistance mutations, preventing the development of *de novo* antibiotic resistance during treatment of infections (19–21). Previous findings showed that Mfd, an RNA polymerase (RNAP)-associated DNA translocase implicated in transcription-coupled nucleotide excision repair, increases mutagenesis and accelerates evolution of drug resistance across highly divergent species, possibly through the formation of R-loops (22–24). We therefore reasoned that directly inhibiting Mfd activity by a small molecule during treatment of infections could decrease mutagenesis, thereby reducing the chances of antibiotic resistance development. In this work, we describe our discovery of a small molecule that modulates the activity of Mfd, demonstrating both that directly targeting bacterial evolvability factors is possible and that this strategy can impede the development of antibiotic resistance by reducing target and non-target-based mutagenesis.

## Materials and methods

### Bacterial culture

*Escherichia coli* NM525 (25), *Salmonella enterica* serovar Typhimurium ST19 (26), *Listeria monocytogenes* 10403S and *S. aureus* (27) were cultured in lysogeny broth (LB), and *Pseudomonas aeruginosa* CF127 (28) in LB with 0.1% Tween 20 (when in liquid media) (Supplementary Table S1). Bacterial plates were grown overnight at 37°C unless otherwise indicated with the following antibiotics when appropriate: 25 μg/ml chloramphenicol, 100 μg/ml carbenicillin or 10 μg/ml tetracycline. When grown in liquid media, cultures were started from single colonies and were grown with aeration (260 rpm).

### Plasmid construction

The *S. enterica mfd* gene along with 200 bp of upstream sequence was amplified with primers HM3564 and HM3565 (Supplementary Table S2), which add NheI and XhoI sites, respectively. pUC19 (NEB) was amplified with primers HM3566 and HM3567 (Supplementary Table S2), which add XhoI and NheI sites. Fragments were digested and ligated according to the manufacturer’s instructions to create pUC19-Mfd.

The NLuc gene was amplified from pNL1 (Promega) with primers HM3568 and HM3569 (Supplementary Table S2). pRGB-KA4 (29) was amplified with primers HM3570 and HM3571 (Supplementary Table S2). Both fragments were joined by Gibson assembly (NEB) according to the manufacturer’s instructions to form pRCB-Nluc.

### High-throughput screen

This screen was performed at Calibr (Scripps Research Institute, San Diego, CA). Δ*mfd E. coli* cells (Supplementary Table S1) (29) with two plasmids, pRCB-NLuc, which expresses the NLuc luciferase protein under a constitutive promoter followed by a *lacO* operator site, and pUC19-Mfd (or pUC19 as a negative control), were grown for ∼24 h in M9 minimal media with 0.2% glycerol as a carbon source at 37°C, shaking in the presence of tetracycline and carbenicillin. Compounds were pre-spotted into 1536-well, white, solid bottom plates (20 μM for primary screen and 40 μM top concentration for dose response) and cells, OD_600_-adjusted in minimal media, were dispensed into these prepared plates. Calmidazolium chloride (20 μM) was used as a stimulator scale reference in lieu of a true activator control and the same volume of dimethyl sulfoxide (DMSO) is used as the neutral control. Following a 4 h incubation at 37°C, the luminescence signal is read using Nano-Glo Luciferase Assay System (Promega) and a microplate reader.

### Preparation of ARM-1

#### Materials

Solvents were either obtained from an MBraun MB-SPS solvent system or freshly distilled (tetrahydrofuran was distilled from sodium benzophenone; toluene was distilled from calcium hydride and used immediately; DMSO was distilled from calcium hydride and stored over 4 Å molecular sieves). Commercial reagents were used as received. The molarity of *n*-butyllithium solutions was determined by titration using diphenylacetic acid as an indicator (average of three determinations).

#### Instrumentation

Semi-preparative reverse-phase high-performance liquid chromatography (HPLC) was conducted on a Waters HPLC system using a Phenomenex Luna 5 μm C18(2) 100A Axia 250 mm × 10.00 mm column or preparative reverse-phase HPLC (Gilson) using a Phenomenex Luna column (100 Å, 50 mm × 21.20 mm, 5 μm C18) with UV/Vis detection. Infrared spectra were obtained as thin films on NaCl plates using a Thermo Electron IR100 series instrument and are reported in terms of frequency of absorption (cm^−1^). ^1^H NMR spectra were recorded on Bruker 400, 500 or 600 MHz spectrometers and are reported relative to deuterated solvent signals. Data for ^1^H NMR spectra are reported as follows: chemical shift (*δ* ppm), multiplicity (s = singlet, d = doublet, t = triplet, q = quartet, p = pentet, m = multiplet, br = broad, app = apparent), coupling constants (Hz) and integration. ^13^C NMR spectra were recorded on Bruker 100, 125 or 150 MHz spectrometers and are reported relative to deuterated solvent signals. Liquid chromatography/mass spectrometry (LC/MS) was conducted and recorded on an Agilent Technologies 6130 Quadrupole instrument.

#### Compound preparation

Compound **1** (Supplementary Figure S1A): (5-Formylfuran-2-yl) boronic acid (1.0 g, 7.15 mmol), 4-bromo-1-iodo-2-methylbenzene (1.63 g, 5.50 mmol), sodium carbonate (2 ml, 2 M solution) and bis(triphenylphosphine) palladium(II) dichloride (193 mg, 0.28 mmol) were added to pressure vessel and dissolved in dimethoxyethane (2 ml) and ethanol (3.5 ml). The solution was sparged with argon for 5 min, and the reaction was then sealed and heated to 65°C for 12 h. The reaction was then cooled to room temperature and diluted into ethyl acetate/H_2_O (30 ml, 1:1). The organic layer was separated, and the aqueous layer was extracted with ethyl acetate (3 × 30 ml). The combined organic layers were dried over MgSO_4_, filtered and concentrated *in vacuo*. The crude product was purified by ISCO column chromatography eluting with 0–40% EtOAc in hexanes to afford the brown solid title compound **1** (1.13 g, 77% yield). ^1^H NMR (400 MHz, CDCl_3_) *δ* 9.68 (s, 1H), 7.67 (d, *J* = 8.4 Hz, 1H), 7.46–7.41 (m, 2H), 7.33 (d, *J* = 3.6 Hz, 1H), 6.74 (d, *J* = 3.6 Hz, 1H), 2.53 (s, 3H) (Supplementary Figure S1A).

Anti-resistance molecule 1 (ARM-1; Supplementary Figure S1B): To a solution of compound **1** (0.5 g, 1.89 mmol) in dichloromethane (8 ml) was added *N*,*N*-dimethylpropane-1,3-diamine (0.25 g, 2.45 mmol). After stirring 2 h at room temperature, sodium triacetoxyborohydride (0.44 g, 2.08 mmol) and acetic acid (0.8 ml) were added to reaction mixture. The reaction mixture was stirred for 16 h at room temperature, quenched with saturated sodium bicarbonate (20 ml) and extracted with dichloromethane (3 × 30 ml). The combined organic phase was dried over MgSO_4_, filtered and concentrated *in vacuo*. The crude product was purified by ISCO column chromatography eluting with 0–70% MeOH in dichloromethane to afford the yellow oil product (0.29 g, 44% yield). ^1^H NMR (400 MHz, CD_3_OD) *δ* 7.63 (d, *J* = 8.4 Hz, 1H), 7.44 (s, 1H), 7.39 (dd, d, *J* = 8.4, 1.6 Hz, 1H), 6.60 (d, *J* = 3.6 Hz, 1H), 6.42 (d, *J* = 3.6 Hz, 1H), 3.83 (s, 2H), 2.67 (t, *J* = 7.2 Hz, 2H), 2.48 (s, 3H), 2.37 (t, *J* = 7.2 Hz, 2H), 2.25 (S, 6H), 1.73 (p, *J* = 7.6 Hz, 2H); LC/MS calculated for C_17_H_23_BrN_2_O [*M* + H]^+^: 351.3; measured: 352.4 (Supplementary Figures S1B and S2).

### Mammalian cell culture

HEK293, HeLa and Caco-2 mammalian cell lines were obtained from the American Tissue Culture Consortium. All cell lines were cultured in Dulbecco’s modified Eagle medium (Gibco) with 10% heat-inactivated fetal bovine serum (HyClone) and 100 U/ml of penicillin–streptomycin (Gibco) at 37°C and 5% CO_2_.

### Translocase assay

**Figure 1.**
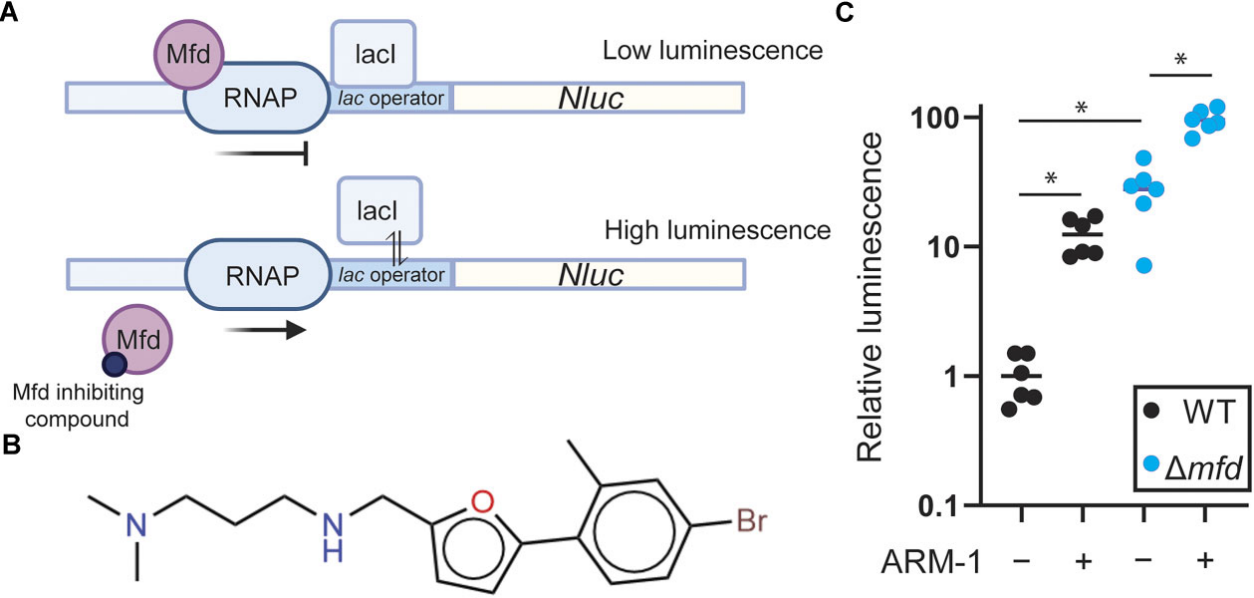
*In vivo* high-throughput screen identifies ARM-1 as an inhibitor of Mfd. (**A**) Schematic of the *in vivo* screen design. When Mfd is present, RNAP complexes stalled at the LacI-bound operator sequence are quickly removed, resulting in little transcription of the *Nluc* gene and low luminescence output. When Mfd is either absent or inhibited, RNAP complexes stalled at the operator are not removed and are able to proceed during temporary dissociation of LacI, resulting in higher levels of luminescent output. (**B**) Structure of ARM-1. (**C**) Normalized luminescence/OD produced by Δ*mfd E. coli* cells containing pRCB-NLuc and either *S. enterica mfd* (wild type, WT) or an empty vector (Δ*mfd*); 100 μM ARM-1 was included when indicated. Horizontal lines represent the average. Statistical significance was determined via Welch’s ANOVA, *P < 0.05; *n* = 6 biological replicates (WT − ARM-1 versus WT + ARM-1, *P* = 0.0027; WT − ARM-1 versus Δ*mfd* − ARM-1, *P* = 0.0122; Δ*mfd* − ARM-1 versus Δ*mfd* + ARM-1, *P* = 0.001).

For Figure 1C, HM3664 (*E. coli* NM525 Δ*mfd* + pRCB-Nluc + pUC19) and HM3419 (*E. coli* NM525 Δ*mfd* + pRCB-Nluc + pUC19-Mfd) were grown overnight in M9 minimal media with 0.2% glycerol as a carbon source with antibiotic selection, in the presence of 100 μM ARM-1 or the appropriate amount of solvent (DMSO), at 37°C, while shaking. OD_600_ and luminescence were both measured the next day using a BioTek Synergy Neo plate reader. Luminescence was generated using the Nano-Glo^®^ Luciferase Assay System (Promega) according to the manufacturer’s instructions. For Supplementary Figure S3, 1 mM isopropyl β-d-1-thiogalactopyranoside (IPTG) was added when indicated.

### *Escherichia coli* Mfd purification

*Escherichia coli* Mfd was purified by growing up *E. coli* cells expressing pCA24N-Mfd (30). Seventy-five milliliters of culture was grown overnight from a single colony in LB + chloramphenicol with agitation at 37°C. The next day 1 l of fresh LB + chloramphenicol was inoculated with 10 ml of overnight culture, incubated with agitation at 37°C. At an OD_600_ of 0.3, 1 mM of IPTG was added and growth was continued for another 4 h. Cells were then centrifuged at 5000 rpm for 10 min and washed with phosphate-buffered saline (PBS). Cell paste was then resuspended in 30 ml HisTrap lysis buffer (50 mM NaPO_4_, pH 8.0; 0.5 mM DTT; 0.5 M NaCl; 5 mM imidazole, pH 8.0) plus 2× Roche inhibitor protease pills and homogenized. Cells were sonicated at midi-tip for 5 min at 50% amplitude with 30 s pulse intervals in an ice bath and then centrifuged at 15 000 rpm for 30 min at 4°C. Supernatant was loaded on a HisTrap column (6 ml) in the same buffer, washed with Ni wash buffer (50 mM NaPO_4_, 300 mM NaCl and 40 mM imidazole, pH 7.4) and then eluted with Ni elution buffer (50 mM NaPO_4_, 300 mM NaCl and 150 mM imidazole, pH 7.4). One 15 ml fraction was collected and dialyzed overnight in a Slide-A-Lyzer (Thermo Scientific) against 1000 ml of TGED buffer (10 mM Tris–HCl, pH 8.0; 5% glycerol; 0.1 mM DTT; 0.1 mM EDTA; 50 mM NaCl). Dialysis material was centrifuged at 15 000 rpm for 30 min at 4°C. Using an AKTA system, supernatant was loaded at 3× Heparin (1 ml) columns (GE) in TGED buffer + 50 mM NaCl buffer at 0.6 ml/min (pressure 0.5 mPa) and eluted by the same buffer with 1 M NaCl in linear gradient from 0% to 100% in 20 column volumes. Fractions were 1 ml during loading and 1 ml during separation. Peaks with a correct molecular weight from Heparin purification were diluted to 50 ml in TGED buffer without NaCl and loaded at MonoQ 5/50 column (GE) in TGED buffer + 50 mM NaCl buffer at 2 ml/min (pressure 3.4 mPa) and eluted by the same buffer with 1 M NaCl in linear gradient from 0% to 100% in 20 column volumes. Fractions were 1 ml during loading and 0.5 ml during separation. Eluate was dialyzed overnight against 1000 ml of 10 mM RA buffer (10 mM HEPES, 1 mM DTT, 50% glycerol, 1 mM EDTA, 500 mM KCl and 40 mM MgCl_2_, pH 8.0) in a Slide-A-Lyzer. The resulting solution was aliquoted in 0.1 ml parts and flash-frozen in liquid nitrogen before being stored at −80°C.

### Mammalian cell cytotoxicity

Cytotoxicity of ARM-1 against HeLa, Caco-2 and HEK293 cells was determined using CellTox Green Cytotoxicity Assay (Promega) according to the manufacturer’s instructions. ARM-1 was diluted in DMSO and applied to the culture such that final concentrations of DMSO were <0.05%. Reported toxicity is relative to DMSO-only control. Cells were incubated with ARM-1 for 24 h prior to addition of reporter dye. Fluorescence was read on a BioTek Synergy Neo plate reader.

### Binding affinity analysis

Microscale thermophoresis was performed on a Monolith NT.115 (NanoTemper). Purified Mfd was His-tag labeled using NanoTemper’s His-Tag Labeling Kit (MO-L018) according to the manufacturer’s protocol. Serial 1:2 dilutions were made of ARM-1 starting with a concentration of 1 mM in PBST buffer for a total of 16 dilutions. Labeled Mfd was then added to each dilution with an end concentration of 50 nM. MST experiments were performed under default settings apart from fluorescence intensity set to 100%. The raw data were imported into Prism 10 software and nonlinear regression was used to find the *K*_D_.

### Computational docking

Coordinates for the crystal structure of *E. coli* Mfd [apo structure, PDB ID 2eyq (29)] and the cryo-EM structure of Mfd bound to DNA and RNAP [holo structure, PDB ID 6x50 (31)] were retrieved from the Protein Data Bank (http://www.rcsb.org) (32). ARM-1 coordinates and parameters were obtained from the ZINC database (https://zinc.docking.org/substances/home/) (33), ZINC4999732, using the following variables: net charge +1, two H-bond acceptors, two H-bond donors and seven rotatable bonds. We used the online version of SwissDock (http://www.swissdock.ch/docking) (34) for computational docking of ARM-1 to Mfd. Separate regions of Mfd, N-terminal (Pro2–Asn352), central (Leu353–Ser999) and C-terminal (Gln1000–Ala1147), were individually submitted for docking to comply with the maximum number of allowed parameters for the computations. Clusters of docking predictions were inspected in UCSF Chimera (35) and the best solutions based on relative energy for each region were used for figure preparation using UCSF Chimera without further refinement.

### NADH-coupled ATPase assay

ATPase assay was measured with an NADH-coupled photometric assay (36). Reactions were performed at 37°C in a 150 μl reaction volume in a 96-well plate. Reactions were performed in repair buffer [40 mM HEPES, pH 8.0, 100 mM KCl, 8 mM MgCl_2_, 4% glycerol (v/v), 5 mM DTT and 100 μg/ml bovine serum albumin] supplemented with 4.4 units of pyruvate kinase, 5.7 units of lactate dehydrogenase, 500 μM phosphoenolpyruvate and 400 μM NADH. Mfd in a final concentration of 50 nM and ARM-1 (aqueous solution, pH 8.0) in varying concentrations were added at least 15 min prior to starting reaction and allowed to incubate on ice. To start the reaction, varying quantities of ATP were added and absorbance at 340 nm was measured every 60 s for 1 h in an Epoch2 microplate spectrophotometer (BioTek).

### Growth curves

A single colony of the indicated species was grown to an OD_600_ of 1–2. The culture was diluted back to an OD_600_ of 0.01 in liquid culture media and grown in an Epoch microplate spectrophotometer (BioTek) at 37°C for 16 h, with 100 μM ARM-1 when indicated. OD_600_ was measured every 10 min.

### RNAP displacement assay

Five nanograms of a ^32^P-labeled, 176 bp polymerase chain reaction fragment containing the promoter of the ampicillin resistance gene from pDR110, a gift from David Rudner, was incubated with 1 unit of *E. coli* RNAP holoenzyme (NEB) for 15 min at 37°C. Then, NTPs were added to a final concentration of 1.7 mM (ATP) or 80 μM (UTP, GTP), as well as 80 μM ApU (Jena Bioscience), and incubated for 15 min at 37°C. Purified *E. coli* Mfd (final concentration of 250 nM) that had been pre-incubated for 10 min at 37°C with 25 μM ARM-1 (aqueous solution, pH 8.0) was added and incubated for 6 min at 37°C. Four microliters of the reaction was loaded into a polyacrylamide gel that had been pre-run for 45 min at 70 V, and run for 55 min at 150 V on ice, using 1× TBE buffer. The products were analyzed by phosphor imaging (GE Healthcare) and quantified using Image Lab 6.0.1.

### Luria–Delbruck fluctuation analysis

Overnight cultures of WT and Δ*mfd S. enterica* were grown from single colonies and back-diluted to an OD_600_ of 0.0005. These cultures were grown to OD_600_ = 0.8–1.0, in the presence and absence of 100 μM ARM-1. One milliliter of this exponential phase culture was then centrifuged at 5000 rpm for 5 min at room temperature, resuspended in 100 μl of 1× Spizizen salts (37) and plated on LB supplemented with 80 ng/ml ciprofloxacin. An additional sample of the same cultures was serially diluted and plated on LB without antibiotic selection to enumerate colony forming units (CFUs). LB + ciprofloxacin plates were incubated at 37°C and LB plates were incubated at 30°C overnight, and all colonies were counted the following morning. Mutation rates were calculated using the Ma–Sandri–Sarkar maximum likelihood method from a minimum of 50 biological replicates per genotype and treatment condition using the FALCOR calculator (38).

### Evolution assays

Evolution experiments were performed for the indicated strains and antibiotics as in (22,27). Overnight cultures, started from a single colony, were back-diluted to OD_600_= 0.005 and used to inoculate a 96-well plate. Cells were grown for 24 h with agitation, at 37°C, in LB with a gradient of concentrations of the indicated antibiotic to select for resistance. Optical densities were subsequently measured in an Epoch2 microplate spectrophotometer (BioTek). Cultures that grew (defined by at least 50% growth relative to LB only) at the highest concentration of antibiotic were passaged into fresh LB with antibiotic in a subsequent plate, again at an OD_600_ of 0.005. A total of five serial passages were performed unless the fastest evolving strains became resistant to the maximum amount of antibiotic that is soluble in LB. For all species, antibiotics were diluted 2-fold down each given row in a 96-well plate. For sequencing of resistance loci from select evolution assays, genomic DNA was extracted from bacterial samples using GeneJet Genomic DNA Purification Kit (Thermo Fisher). Samples were Sanger sequenced by Genewiz using custom primers (Supplementary Table S2).

### Mutagenesis measurements after epithelial cell infection

Sixteen hours before the infection, 5 million HeLa cells were seeded into 10 cm plates and incubated overnight in full media without antibiotics. The next morning, a single *S. enterica* colony from strain HM1996 (WT) or HM3429 (Δ*mfd*) was inoculated into 20 ml of LB and grown at 37°C while shaking to an OD_600_ of 0.5. The bacteria were then washed twice with tissue culture grade 1× PBS and resuspended in 2 ml of full media without antibiotics. Bacteria were then applied to the HeLa cells at a multiplicity of infection of 100:1 (125 μl of the cell suspension) and allowed to invade for 60 min at 37°C and 5% CO_2_. After 60 min, the medium was removed, cells were washed with PBS, and fresh medium containing 50 μg/ml gentamycin and 5 μM ARM-1 or the appropriate volume of vehicle (DMSO) was applied to the HeLa cells. This concentration is well below the IC_50_ of this compound in the three human cell lines tested (Supplementary Figure S4). After 24 h of infection, HeLa cells were washed with 1× PBS and lysed with 1 ml of PBS + 1% Triton X-100 for 5 min at room temperature. A small portion of lysate was serially diluted and plated on M9 minimal + 0.4% glycerol agar and grown for 48 h at 30°C for CFU enumeration. The remaining volume was plated on LB + 2 μg/ml 5-fluorouracil and grown for 48 h at 37°C to determine mutation frequency. Mutation frequency is reported as the ratio of number of colonies that grew on selection plates to the number of viable bacterial cells in each lysate.

### Whole genome sequencing and variant calling

Genomic DNA was extracted from evolution assay samples using GeneJet Genomic DNA Purification Kit (Thermo Fisher). Libraries were prepared using Nextera Library Prep Kit (Illumina) and sequenced on a NovaSeq 6000 (Illumina) according to the following specifications: 150PE reads and minimum 150× coverage. Raw reads were processed using fastp for quality control and trimming (39), and then mapped to the appropriate genome using bowtie2 and samtools (40–42). To identify variants, bcftools (42) was used to compare each experimental sample to a WT control processed in parallel. We selected mutations in protein-coding genes that were present in the evolved but not in the parental (unevolved) strain and had a bcftools quality >200. We excluded 1 bp insertions or deletions in homopolymers (four or more identical nucleotides in a row).

### Statistical analysis

All statistical analysis was performed using GraphPad Prism 10.

## Results

### An *in vivo* high-throughput screen identified novel Mfd-inhibiting compounds

Based on genetic analyses, we previously proposed Mfd as a target for inhibiting the rate of antibiotic resistance development (22). To pursue that strategy, we conducted an *in vivo* high-throughput screen of roughly 250 000 compounds. To identify Mfd-specific inhibitors in a biologically relevant context, we modified a previously described roadblock repression assay, which can be used to monitor Mfd-dependent RNAP displacement activity (Figure 1A) (43). The advantages of performing this screen *in vivo* are 2-fold: (i) It maintains all relevant protein interactions that are required for Mfd’s pro-mutagenic activity, such as its interaction with RNAP and the nucleotide excision repair protein UvrA (22), and (ii) it will only identify small molecules that are able to permeate bacterial cells and have little or no bactericidal activity.

For the screen, we used Δ*mfd E. coli* cells containing a reporter system encoded on two different plasmids. One plasmid contains a lac operator (*lacO*) encoded directly upstream of the gene that codes for the NanoLuc protein, a small luciferase that can be used to produce luminescence (44), under the control of a constitutive promoter. The second plasmid includes the *mfd* gene from *S. enterica* serovar Typhimurium ST19 with its endogenous promoter. The LacI protein bound to *lacO* blocks RNAP and is therefore a barrier to transcription, inhibiting the production of luminescence; the stalled RNAP is quickly recognized by Mfd and is pushed off the DNA. However, in the absence of Mfd, the stalled RNAP is able to proceed and transcribe at a significant rate, as LacI ‘breathes’ on and off DNA and temporarily dissociates from the operator sequence (45), ultimately leading to transcription and an increase in luminescence (Figure 1A). To look for Mfd inhibition, we measured relative luminescence in the presence of each compound. To control for off-target effects, we assayed strains carrying plasmids that did not contain the *mfd* gene. In addition, we tested each compound for its toxicity to human cell lines. Positive hits from this system are those compounds that result in high luminescence relative to vehicle (DMSO) only when Mfd is present, and do not show high bactericidal or cytotoxic effects.

This screen led to the identification of 40 compounds that showed properties consistent with Mfd inhibition. After validation of the screen using this translocase assay, analysis of the chemical structures and commonalities between them, and testing for binding to Mfd, we ended up with 10 lead compounds of interest. Here, we present the biochemical and biological effects of one of them, which we have termed ARM-1. The chemical structure of ARM-1 is shown in Figure 1B.

### Synthesis and biochemical characterization of ARM-1

We synthesized ARM-1 to >99% purity following an original synthesis strategy (see the ‘Materials and methods’ section; Supplementary Figures S1 and S2). We used this preparation of the compound to validate the result of the screen using the roadblock repression assay described above and we observed a 13-fold increase in luminescence in cells expressing Mfd that had been grown in the presence of ARM-1 (Figure 1C). We also observed a small (3.5-fold) increase in luminescence by ARM-1 in cells containing an empty vector (Δ*mfd*), suggesting a small Mfd-independent effect of the compound, most likely on RNAP. However, and due to the difference in the effects of ARM-1, we reasoned that Mfd is this compound’s main target and therefore continued with its characterization. As a control, and to determine the dynamic range of the assay, we performed the assay in the presence of 1 mM IPTG, which prevents the binding of the LacI protein to the *lacO* sequence, therefore removing the block to transcription and allowing for maximal expression from the reporter. As expected, we observed even larger increases in luminescence when cells were incubated in the presence of IPTG (Supplementary Figure S3). In addition, we confirmed that ARM-1 does not have a strong cytotoxicity to human cells (Supplementary Figure S4).

**Figure 2.**
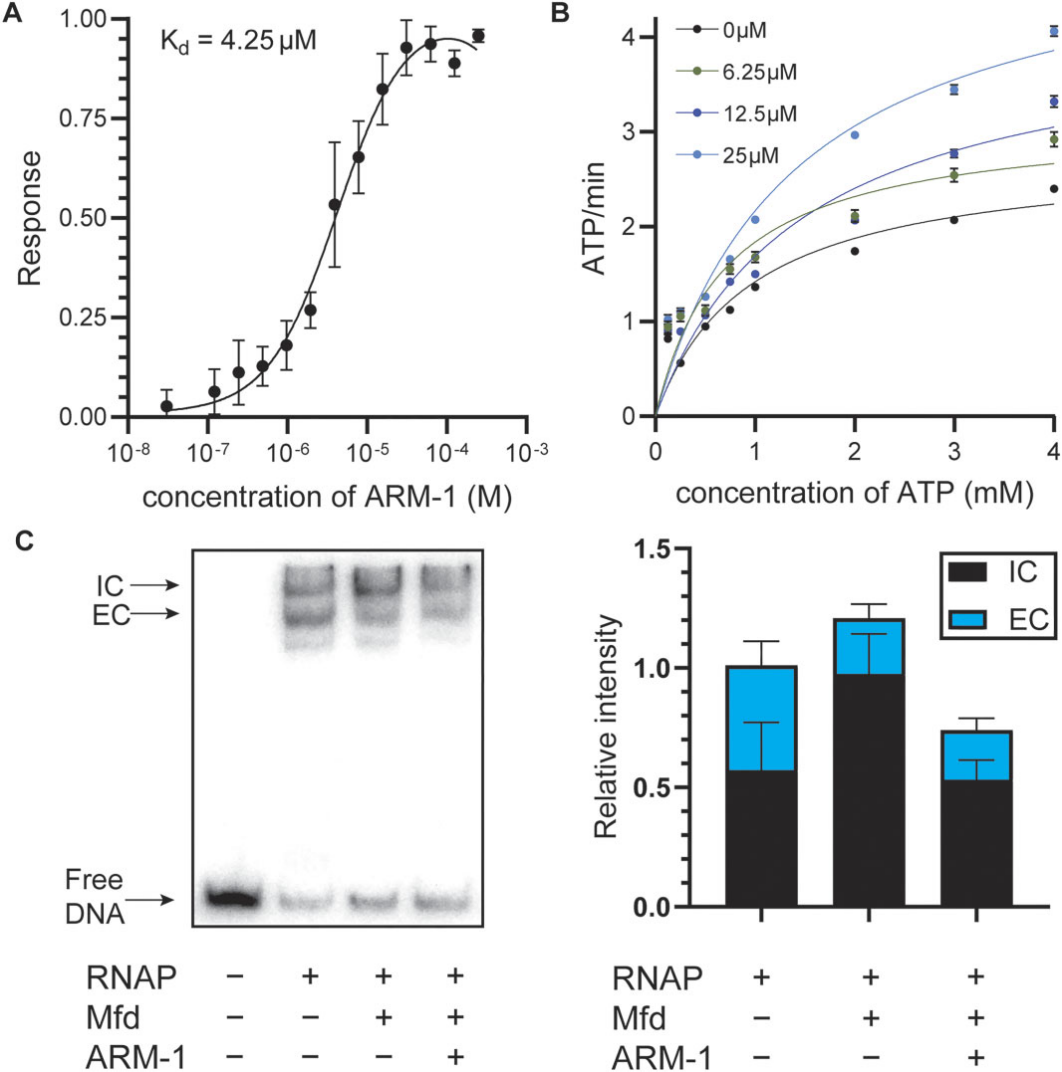
ARM-1 modulates Mfd’s enzymatic activity. (**A**) *K*_D_ of ARM-1 was determined using microscale thermophoresis. Values represent the average of three replicates except for the highest concentration of ARM-1 (*n* = 2). Error bars represent the standard error of the mean (SEM). (**B**) NADH-coupled ATPase assay. Absorbance at 340 nm by NADH is used to monitor the reaction. Data shown represent the average of two replicates except for 0 μM ARM-1 (*n* = 6). Error bars represent the SEM. (**C**) Transcription roadblock assay. RNAP was stalled by CTP starvation after which Mfd (pre-incubated with 25 μM ARM-1 when indicated) was added and allowed to remove stalled RNAP for 6 min. Representative gel as well as the average initiation complex (IC) and elongation complex (EC) intensity of five experiments is shown. Error bars represent the SEM.

Consistent with its inhibition of Mfd activity, ARM-1 binds to Mfd with a *K*_D_ of 4.25 μM, as determined by microscale thermophoresis (Figure 2A). This is an acceptably tight binding affinity for a lead compound prior to medicinal chemistry optimization.

**Figure 3.**
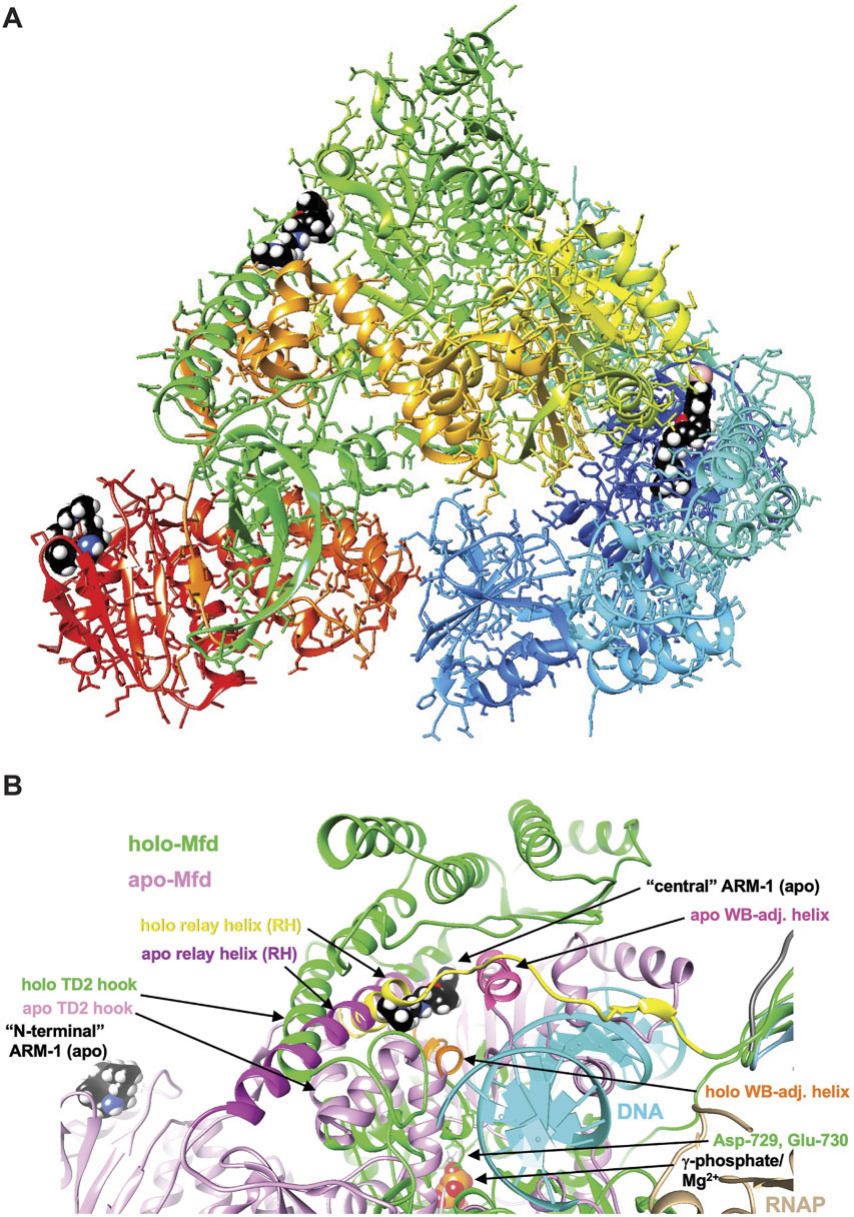
ARM-1 binding sites on *E. coli* Mfd from computational docking. (**A**) Overall view of the crystal structure of *E. coli* Mfd (PDB ID 2eyq, apo form), with potential ARM-1 (shown in space filling mode with black carbon atoms) binding sites in the N-terminal UvrB homology domain (righ part of the protein, in blue), and central (top part, in green) and C-terminal regions (left part, in red). (**B**) Overlay of the Mfd apo (PDB ID 2eyq; pink) and holo structures (PDB ID 6x50; green), affording a close-up view of the first two binding sites in the Mfd apo structure: ‘N-terminal’ and ‘central’. The ARM-1 molecule visible in the center is lodged between the RH and WB-adjacent helix that are colored in magenta and dark pink, respectively, in the apo-Mfd structure. This binding mode of ARM-1 potentially precludes unraveling of RH and a shift by the WB-adjacent helix (colored in yellow and orange, respectively) as seen in Mfd holo structure bound to RNAP and DNA. Selected regions and residues are labeled and ATP/Mg^2+^ and RNAP are visible near the lower edge of the image, middle and right, respectively.

Since ARM-1 binds to Mfd *in vitro*, we performed computational docking between ARM-1 and Mfd using SwissDock (34). We ran the *E. coli* Mfd apo structure [PDB ID 2eyq (29)] in three parts: N-terminal (Pro2–Asn352), central (Leu353–Ser999) and C-terminal (Gln1000–Ala1147), since Mfd is too large for this software, and we obtained ARM-1’s structure from ZINC20 (33). We found possible ARM-1 binding sites in all three parts, with the interaction energies being −15.7, −19.4 and −10.4, respectively (Figure 3A). The N-terminal binding site is to the UvrB homology module (Supplementary Figure 3A and B), which binds to UvrA to recruit the NER machinery (46), providing a possible mechanism of action for ARM-1. In addition, the central region binding site is adjacent to the relay helix (RH) and the Walker B motif (WB)-adjacent helix (Figure 3B). RH and WB-adjacent helix display complete unraveling and a large shift, respectively, upon binding by Mfd to RNAP and DNA to form the holo structure [PDB ID 6x50 (31)] (Figure 3B). ARM-1 binding could potentially prevent these conformational rearrangements, thereby affecting Mfd’s function. A structural overlay for Mfd in the apo and holo forms is shown in Supplementary Figure S5.

We then set out to determine how ARM-1 is inhibiting Mfd. Mfd translocates along double-stranded DNA and dislodges stalled RNAP complexes (47,48). As both processes are dependent on Mfd’s ability to hydrolyze ATP (31,49), we directly examined the effect of ARM-1 *in vitro* on Mfd’s ATPase activity. Surprisingly, we did not observe a decrease in Mfd’s ability to hydrolyze ATP in the presence of ARM-1, which we measured by using an NADH-coupled assay (Figure 2B and Table 1). If anything, we observed an increase at the highest concentration tested (Figure 2B and Table 1). We showed that ARM-1 does not produce signal at 340 nm, the wavelength used to measure ATP hydrolysis in this assay, further confirming the reliability of these measurements (Supplementary Figure S6A). Therefore, we concluded that a direct effect on ATP hydrolysis is not a primary avenue by which ARM-1 modulates Mfd activity.

**Table 1.** Kinetic constants of Mfd ATPase activity determined using Michaelis–Menten modeling

	*V*_max_		*K*_m_		
[ARM-1]	Mean	SEM	Mean	SEM	*R* ^2^
0 μM	2.771	0.343	0.954	0.319	0.8941
6.25 μM	3.135	0.338	0.706	0.228	0.8806
12.5 μM	4.170	0.711	1.470	0.583	0.8858
25 μM	5.216	0.631	1.408	0.403	0.9411

To further investigate the mechanism by which ARM-1 affects Mfd activity, we performed a slightly modified version of an established *in vitro* RNAP displacement assay (50). In this assay, *E. coli* RNAP holoenzyme is stalled by CTP starvation on a short, radiolabeled DNA fragment containing a constitutively active promoter. The first template guanine encoded on this DNA fragment is 21 nucleotides downstream of the promoter. In the absence of CTP, RNAP stalls at this location, and can be removed by Mfd. Performing an electrophoretic mobility shift assay with the products of this transcription roadblock reaction reveals two DNA–protein complexes: a slower migrating IC and a faster migrating EC at roughly the same levels (Figure 2C and Supplementary Figure S6B) (49,50). The addition of purified Mfd removes the stalled RNAP from the EC and allows it to re-bind the promoter and form an IC, which results in an increase in the ratio of ICs to ECs (Figure 2C and Supplementary Figure S6C). When we pre-incubated Mfd with 25 μM ARM-1, though we still observed a decrease in the amount of EC when compared to the reaction without Mfd (i.e. RNAP was still being displaced), we no longer observe the corresponding increase in ICs (Figure 2C and Supplementary Figure S6C). This suggests that ARM-1 inhibits Mfd’s function not by interfering with its ability to remove stalled RNAPs from DNA, a function that depends on Mfd’s ATPase activity, but by preventing RNAP from re-binding the promoter (Supplementary Figure S6C). This is consistent with the fact that Mfd seems to be unable to bind to RNAPs once they are bound to promoters (51). Importantly, when we performed these reactions in the absence of Mfd, we did not observe a change in the ratio of IC to EC, indicating that ARM-1 does not have a significant effect on the *in vitro* activity of RNAP in the absence of Mfd (Supplementary Figure S6B). Considering these results together with the ATPase activity assays, we propose that Mfd displaces RNAP not only through enzymatic activity, but also through an allosteric mechanism, which is common to transcription terminators such as Rho (52–54).

### ARM-1 reduces mutagenesis and the evolution of antibiotic resistance

**Figure 4.**
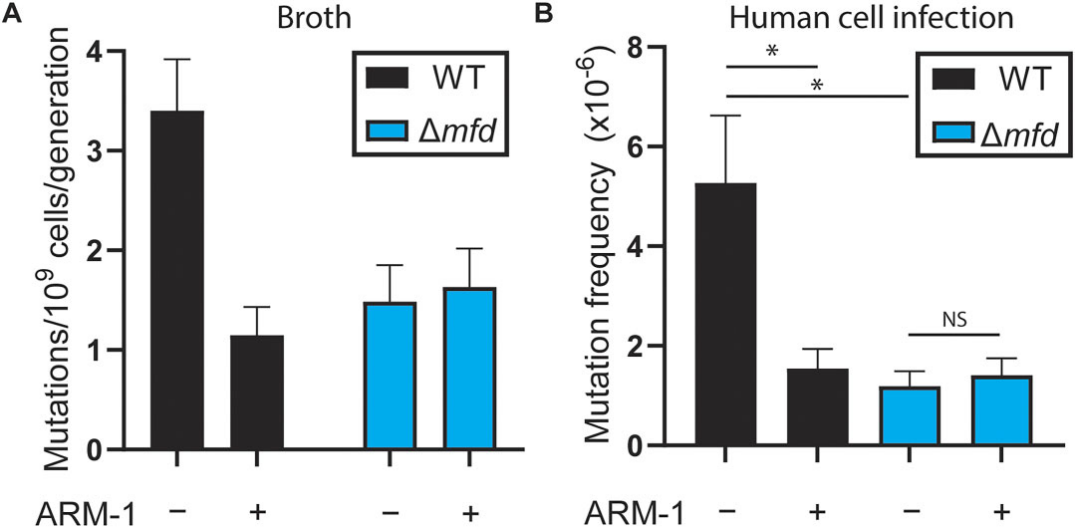
ARM-1 reduces mutagenesis. (**A**) Mutation rates of *S. enterica* strains measured using ciprofloxacin; *n* = 64 (WT − ARM-1), 64 (WT + ARM-1), 52 (Δ*mfd* − ARM-1) and 52 (Δ*mfd* − ARM-1). Cells were grown in the presence of 100 μM ARM-1 when indicated. Error bars represent 95% confidence intervals. (**B**) Mutation frequency after infection of human cells measured using 5-fluorouracil; *n* = 30 (WT − ARM-1), 24 (WT + ARM-1), 18 (Δ*mfd* − ARM-1) and 18 (Δ*mfd* − ARM-1). Cells were grown in the presence of 5 μM ARM-1 when indicated. Error bars represent the SEM. Statistical significance was determined via ordinary one-way ANOVA, *P < 0.05.

Using Luria–Delbruck fluctuation analyses (55), we previously showed that the absence of Mfd leads to decreased mutation rates due to its role in transcription-coupled nucleotide excision repair (22,27). To determine whether ARM-1 is inhibiting Mfd, we measured its impact on mutagenesis. We observed that ARM-1 treatment leads to a 3-fold reduction in WT *S. enterica* mutation rates, but not in Δ*mfd S. enterica* cells, consistent with direct inhibition of Mfd (Figure 4A). Our lab has previously shown that the mutagenic effect of Mfd is conserved during bacterial infection of eukaryotic cells (22). To determine whether ARM-1 can also reduce mutagenesis during infection, we infected HeLa cells with WT and Δ*mfd S. enterica*, and measured mutation frequency through acquisition of 5-fluorouracil resistance (56). As anticipated, we observed a reduction in the mutation frequency when ARM-1 was added to the culture media only in WT *S. enterica* cells (Figure 4B).

**Figure 5.**
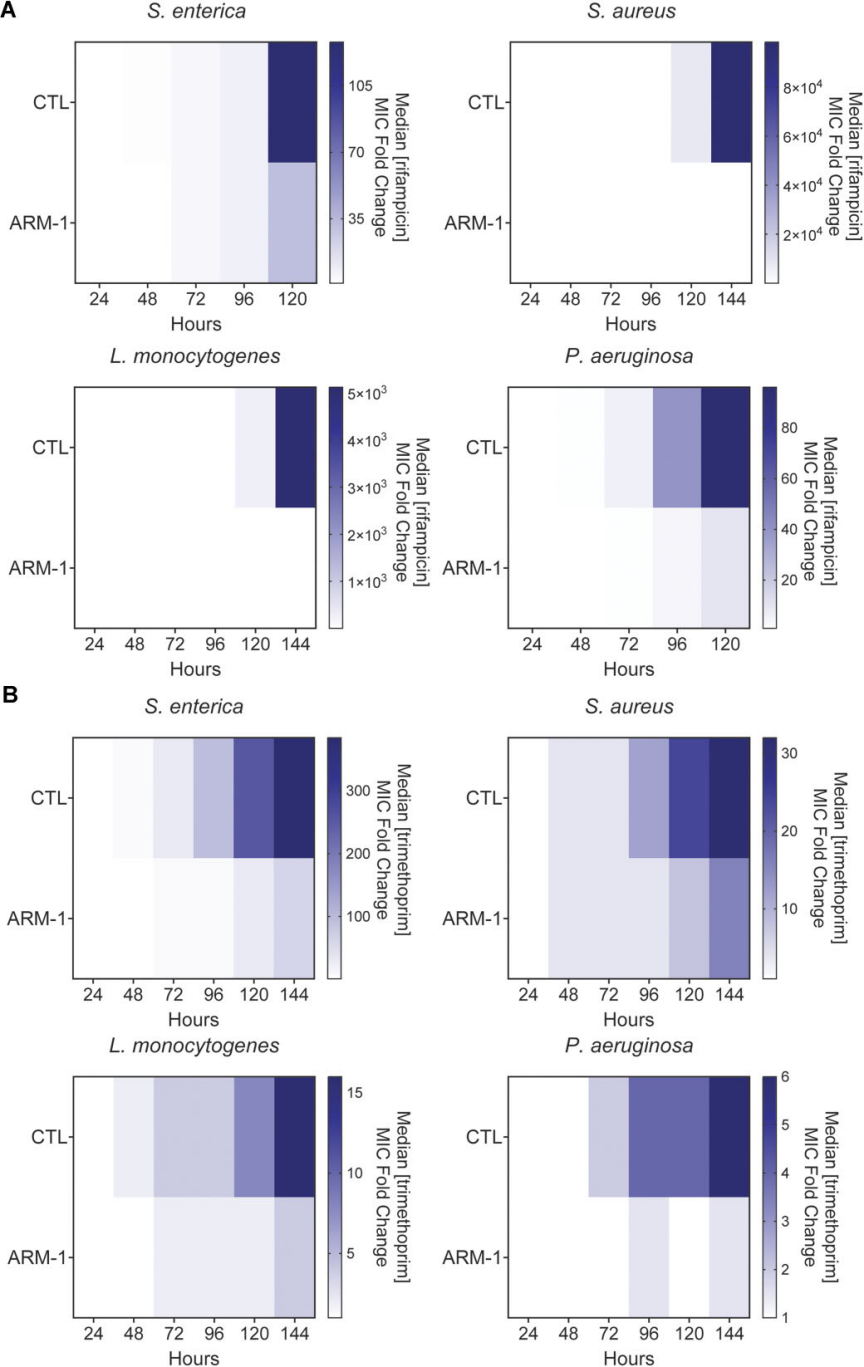
ARM-1 inhibits the evolution of antibiotic resistance. Evolution of indicated species against rifampicin (**A**) and trimethoprim (**B**) in the presence of 100 μM ARM-1. Heatmaps show median MIC at the indicated time points. Data for strain/antibiotic combination presented are the result of at least eight biological replicates.

To determine whether the reduction in mutagenesis by ARM-1 translates into inhibition of drug resistance, we examined the effect of ARM-1 on the evolution of antibiotic resistance across diverse pathogens. We observed a significant reduction in resistance development when bacteria were treated with ARM-1 compared to those that were untreated, in all species and antibiotics tested. This effect was consistent even in strains of bacteria already carrying resistance mutations to other antibiotics, as the strain of *S. aureus* we used is resistant to penicillin, oxacillin and erythromycin (27). In *S. enterica*, challenged with the transcription inhibitor rifampicin or the folate synthesis inhibitor trimethoprim, we observed a significant reduction in the increase of the median minimum inhibitory concentration (MIC) between untreated and treated conditions in these experiments (Figure 5A and B). This pattern was also observed in *S. aureus*, *L. monocytogenes* and *P. aeruginosa*, with ARM-1 treatment resulting in >1000, >600 and >9 reduction in resistance to rifampicin, respectively (Figure 5A). We observe very similar results when these bacteria are treated with trimethoprim (Figure 5B). At the concentration used, ARM-1 did not show any effect on the growth rate of these species, although a slight increase in the lag phase can be seen for *S. aureus* and *L. monocytogenes* (Supplementary Figure S7A–D). This indicates that our results are not simply attributable to growth defects. As previously shown (22), sequencing of relevant resistance loci over the course of treatment confirmed that resistance development correlates with the appearance of mutations in relevant genes that have been shown to lead to resistance (Tables 2–5) (57–59). Populations of cells that were treated with ARM-1 accumulated less mutations compared to untreated cells over the course of the experiment (Tables 2–5).

**Table 2.** Amino acid mutations in the *rpoB* gene of *S. aureus* during rifampicin treatment at the indicated time, with and without ARM-1 treatment

−ARM-1					+ARM-1				
		D471G	D471G	D471G					
	S529L	S529L	S529L, D471G	S529L, D471G					S486L
	D471N	D471N	D471N	D471N			R484C	R484C	R484C
	R484C	R484C	R484C, I527L	R484C, I527L			I527L	I527L	I527L
I527M	I527M	I527M, Q468K	I527M, Q468K	I527M, Q468K			D471G	D471G	D471G, L466S
A430V	A430V	A430V, Q468L	A430V, Q468L	A430V, Q468L		A430T	A430T	A430T	A430T, S417P
S464T	S464T, D471R	S464T, D471R	S464T, D471R	S464T, D471R	A430V	A430V	A430V	A430V	A430V, H481Y
48	72	96	120	144	48	72	96	120	144
Time (h)					Time (h)				

**Table 3.** Amino acid mutations in the *rpoB* gene of *L. monocytogenes* during rifampicin treatment at the indicated time, with and without ARM-1 treatment

−ARM-1					+ARM-1				
			Q470R	Q470R					
		S488L	S488L	S488L					
	H483L	H483L	H483L	H483L					G478D
	Q470R	Q470R	Q470R	Q470R				H483L	H483L
48	72	96	120	144	48	72	96	120	144
Time (h)					Time (h)				

**Table 4.** Amino acid mutations in the *folA* gene of *S. enterica* during trimethoprim treatment at the indicated time, with and without ARM-1 treatment

−ARM-1					+ARM-1				
				F153S, D27E					
			D27E	D27E					
	P21L	P21L	P21L	P21L					
	F153S	F153S	F153S	F153S					F153S
	F153S	D27E, F153S	D27E, F153S	D27E, F153S				P21L	P21L
48	72	96	120	144	48	72	96	120	144
Time (h)					Time (h)				

**Table 5.** Amino acid mutations in the *folA* gene of *S. aureus* during trimethoprim treatment at the indicated time, with and without ARM-1 treatment

−ARM-1					+ARM-1				
			F99Y	F99Y					
		F99Y	F99Y	F99Y					
		F99Y	F99Y	F99Y					
	F99Y	F99Y	F99Y	F99Y				L41F	L41F
48	72	96	120	144	48	72	96	120	144
Time (h)					Time (h)				

If a compound is truly effective at inhibiting a mutagenic mechanism underlying resistance development, bacteria should not acquire mutations in the target gene as a consequence of exposure to the compound. Even though ARM-1 does not impact growth rate at the concentrations used in all other experiments (Supplementary Figure S7A–D), when used at higher concentrations, ARM-1 becomes toxic (Supplementary Figure S7E and F). This property allowed us to address the question of whether cells can become resistant to ARM-1 itself. To test this hypothesis, we repeatedly challenged *S. enterica* and *S. aureus* (representative Gram-negative and Gram-positive pathogens) with increasing concentrations of ARM-1. Over the course of 60–70 generations (196 h), we found that, remarkably, the MIC remains constant in both species tested (Supplementary Figure S7E and F).

In addition, it is vital to determine whether target or non-target mutations (i.e. mutations in Mfd or other relevant genes) arise during treatment at sublethal concentrations of ARM-1. We performed whole genome sequencing of six biological replicates of *S. enterica* and *S. aureus* exposed to 100 μM ARM-1 for 70 generations. We did not observe any mutations in protein-coding genes in *S. enterica*, and only two mutations in *S. aureus*, in *SAOUHSC*_*01556* and *yjbH*, two genes that have not been linked to antibiotic resistance or DNA repair (Table 6) (60). This is consistent with a lack of selection due to the presence of ARM-1. Moreover, almost all mutations (10/12) that arose when samples were treated with ARM-1 in combination with an antibiotic are related to resistance specifically to that antibiotic (Table 6) (22,57,58,61). The two exceptions to this (*murC* and *serB* in *S. enterica* exposed to rifampicin) (Table 6) have also not been linked to DNA repair, suggesting that they are passenger mutations and not related to ARM-1 treatment. These data further support that ARM-1 is effective at inhibiting bacterial mutagenesis and can be used in combination with existing antibiotics to limit resistance development without exerting any additional selection pressure on the population.

**Table 6.** Mutations in protein-coding genes in *S. enterica* and *S. aureus* after 6 days of ARM-1 treatment with and without the indicated antibiotics (ND: none detected)

Species	Treatment	Replicate	Mutations
*Salmonella enterica*	ARM-1	1	ND
		2	ND
		3	ND
		4	ND
		5	ND
		6	ND
	Rif + ARM-1	1	ND
		2	D147A (*murC*) I572L (*rpoB*) S82A (*serB*)
		3	I572N (*rpoB*)
	Trim + ARM-1	1	M199T (*cpxR*)
		2	M199T (*cpxR*)
		3	Q34I (*ygdP*)
*Staphylococcus aureus*	ARM-1	1	ND
		2	ND
		3	N90I (*SAOUHSC*_*01556*)
		4	E183* (*yjbH*)
		5	ND
		6	ND
	Rif + ARM-1	1	Q137L (*rpoB*) I527L (*rpoB*)
		2	L466S (*rpoB*) D471G (*rpoB*)
		3	S486L (*rpoB*)
	Trim + ARM-1	1	ND
		2	ND
		3	ND

## Discussion

Antibiotic resistance is poised to become one of the main public health problems of the 21st century (1–3). Soon after an antibiotic is used in the clinic, bacteria evolve resistance, making the search for new antibiotics alone insufficient (5). Stopping the evolution of antibiotic resistance is therefore key to fighting the war against antibiotic resistance development.

Here, we describe the first anti-evolution compound that directly inhibits a transcription-associated mutagenic bacterial protein, ARM-1, a small molecule that inhibits the evolution of antibiotic resistance by targeting the RNAP-associated translocase Mfd. We had previously proposed Mfd as a target for the inhibition of antibiotic resistance as it promotes mutagenesis and is required for rapid resistance development to antibiotics with different modes of action across highly divergent bacterial species (22). Through an *in vivo* screen, we identified and then characterized the effects of the lead compound ARM-1 on Mfd both *in vitro* and *in vivo*. We observed that ARM-1 affects Mfd’s function without significantly affecting its ATPase activity. These observations suggest that in addition to its ATPase activity, Mfd also harbors an allosteric mechanism that is essential for its RNAP removal and recycling, which is consistent with the ARM-1 binding sites predicted by computational docking. This finding makes a valuable contribution to the field and provides a new tool (ARM-1) to further study the mechanism by which Mfd removes RNAP from DNA.

It is important to note that previous attempts have been made to utilize small molecules to inhibit the evolution of antibiotic resistance. Edaravone, a molecule used in the treatment of amyotrophic lateral sclerosis, has been shown to be able to decrease the mutagenesis associated with subinhibitory concentration of one antibiotic—ciprofloxacin (62). Ciprofloxacin leads to the generation of a subpopulation of cells that are under high oxidative stress, triggering the pro-mutagenic σ^S^ general stress response, and edaravone works as an antioxidant to counter this phenomenon (62). Importantly, ARM-1’s activity is not limited to antibiotics that lead to an increase in reactive oxygen species (ROS), as it inhibits the evolution of resistance against rifampicin, which does not increase the amount of ROS in bacterial cells (27,63). Furthermore, given that macrophages use ROS to fight infections, treatment with a drug such as edaravone might have adverse effects such as decreasing the ability of the host to kill the pathogen.

Targeting the SOS response has also been proposed as a way to decrease mutagenesis leading to antibiotic resistance (16,17,64). This has been attempted for two key factors in the SOS response, LexA and RecA (65–67). However, effectively targeting LexA with a competitive inhibitor has proved to be challenging (18). In the case of RecA, bacteria lacking this protein display severe growth defects relative to WT (68,69). This phenotypic defect would quickly lead to the appearance of resistance to any RecA inhibitors, which is not optimal. As our data show, this is not the case for Mfd as Δ*mfd* cells show no growth defect (22), and we have not detected resistance development to ARM-1 in evolution assays.

Resistance can also spread rapidly through diverse populations of pathogens through horizontal gene transfer (HGT) (70). However, even in the case of HGT, bacteria can acquire mutations that generate a diverse array of functions within the resistance gene carried on mobile elements (18). It is important to consider that the diversity of genetically transferable elements, whether plasmid or chromosomally encoded, mostly occurs through mutagenesis, and will be enhanced upon exposure to antibiotics due to positive selection for the resistant variants. This theory is supported, for example, by the fact that since the widespread use of penicillin began in the 1940s, over 2000 different β-lactamases have been identified (71). This variation has been attributed to hundreds of point mutations within the β-lactamase gene (71). While HGT may be a proximal cause of resistance spread within a population, mutations are still the cause of a significant proportion of antibiotic resistance development through this mechanism. Therefore, an anti-evolution drug that reduces mutagenesis could also help reduce HGT-related antibiotic resistance development.

ARM-1 decreases mutagenesis that drives antibiotic resistance development, both in pure bacterial culture and during eukaryotic cell infection. Accordingly, it also decreases the rate of antibiotic resistance development in all medically relevant pathogenic species tested, including those that already carry resistance mutations, such as multidrug-resistant strains of *S. aureus*. Additionally, the findings described here, the discovery of ARM-1, show that, in general, it is possible to inhibit evolution through a small molecule. The development of such compounds into a drug that can be used in the clinic could be a game changer in the prevention of antibiotic resistance development during the treatment of infections.

## Supplementary Material

ugae001_supplemental_file

## Data Availability

All raw fastq files from high-throughput sequencing data have been deposited at the National Center for Biotechnology Information (NCBI) Sequence Read Archive (SRA) (accession number PRJNA1032096).
