# Supplementary material for: A small molecule that inhibits the evolution of antibiotic resistance
Source: NAR Mol Med. 2024 Jan 23;1(1):ugae001. doi: 10.1093/narmme/ugae001 (PMC11188740; doi:10.1093/narmme/ugae001)
Supplement: ugae001_supplemental_file [file ugae001_supplemental_file.pdf]

SUPPLEMENTARY MATERIAL

Supplementary Figures

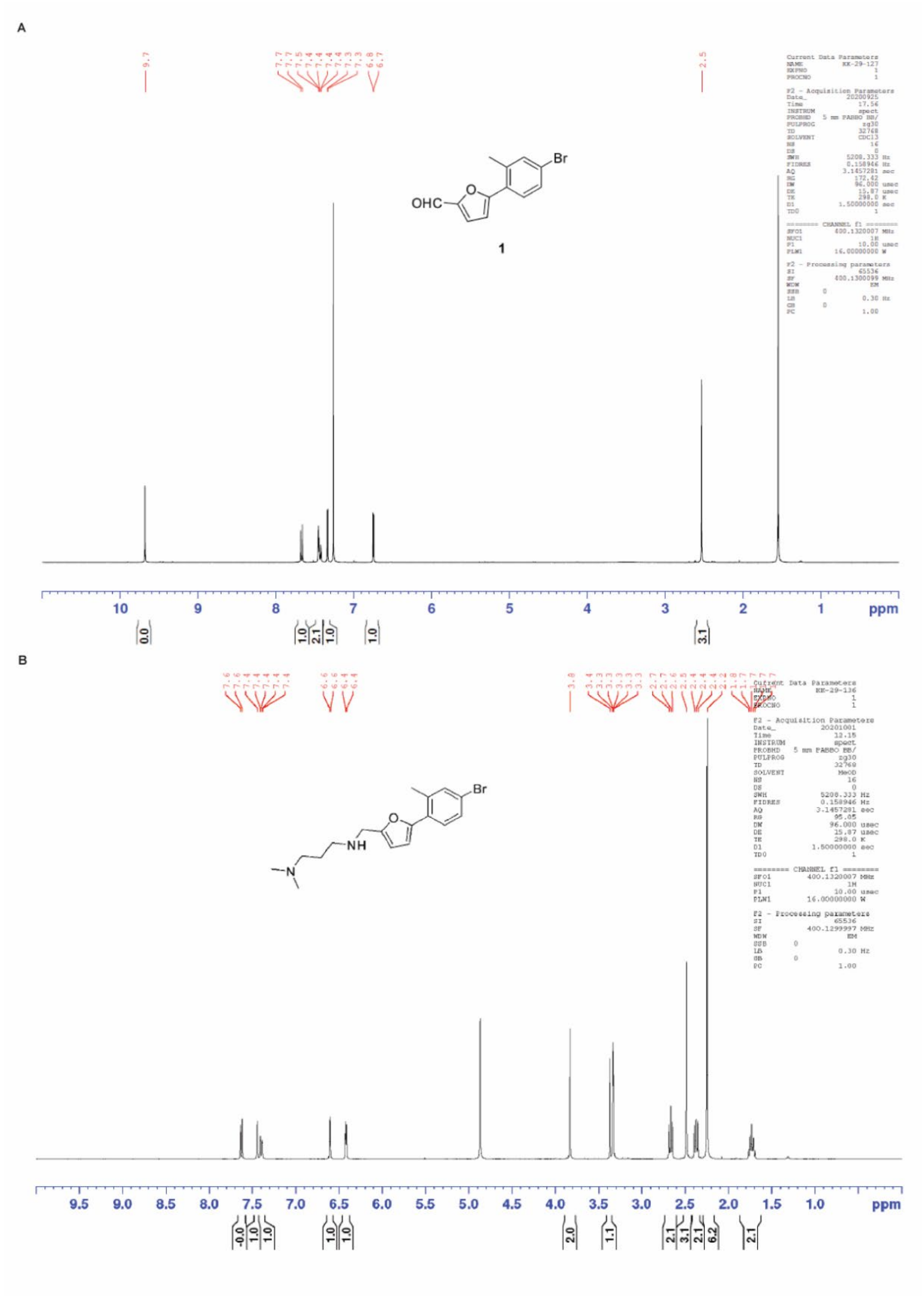

Figure S1: NMR spectrum of Compound 1 (A) and ARM-1 (B)

Peak #	Time	Area % UV215	Area % UV254	Area % TIC(+)	BPM	Area Abs UV215	Area Abs UV254	Area Abs TIC(+)
1	0.112	0.0	19.0	2.3	214.2	0	103.26	96822.67
2	0.841	100.0	81.0	97.7	351.3	641.83	441.29	4030277.75
3	0.919	0.0	0.0	0.0	351.3	0	0	0

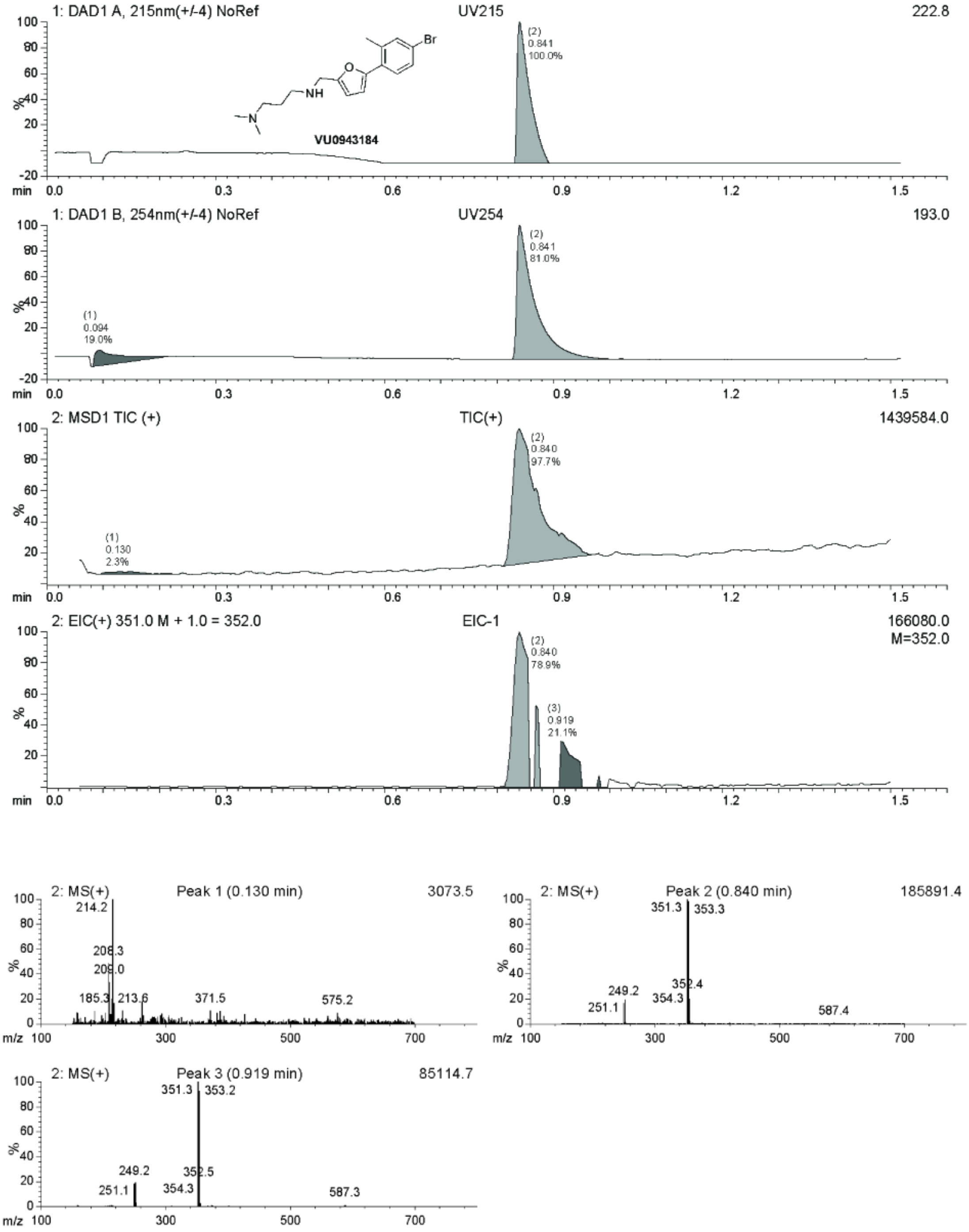

Figure S2: ARM-1 Liquid Chromatography with Tandem Mass Spectrometry (LCMS)

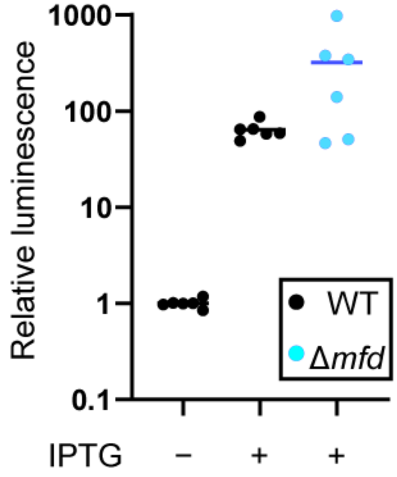

Figure S3: Normalized luminescence/OD produced by Δ*mfd E. coli* cells containing pRCB-NLuc and either *S. enterica mfd* (WT) or an empty vector (Δ*mfd*). 1 mM IPTG was included when indicated. Lines represent the average.

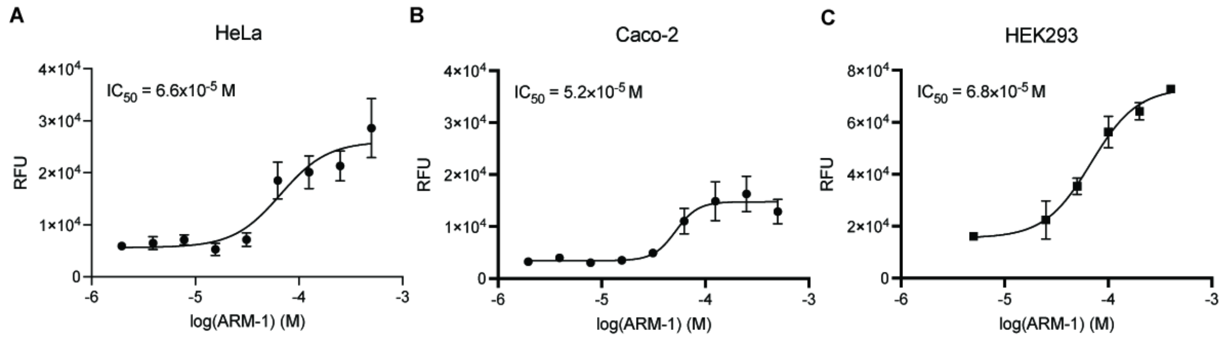

Figure S4: Cytotoxicity of ARM-1 against the indicated mammalian cell lines. Toxicity was determined using Promega CellTox reagents following 8 hours of exposure of HeLa, Caco-2, and HEK293 cells to varying concentrations of ARM-1. Relative fluorescent units (RFUs) reported relative to solvent and no substrate controls. n=4 (HeLa and Caco-2) or 2 (HEK293) biological replicates.

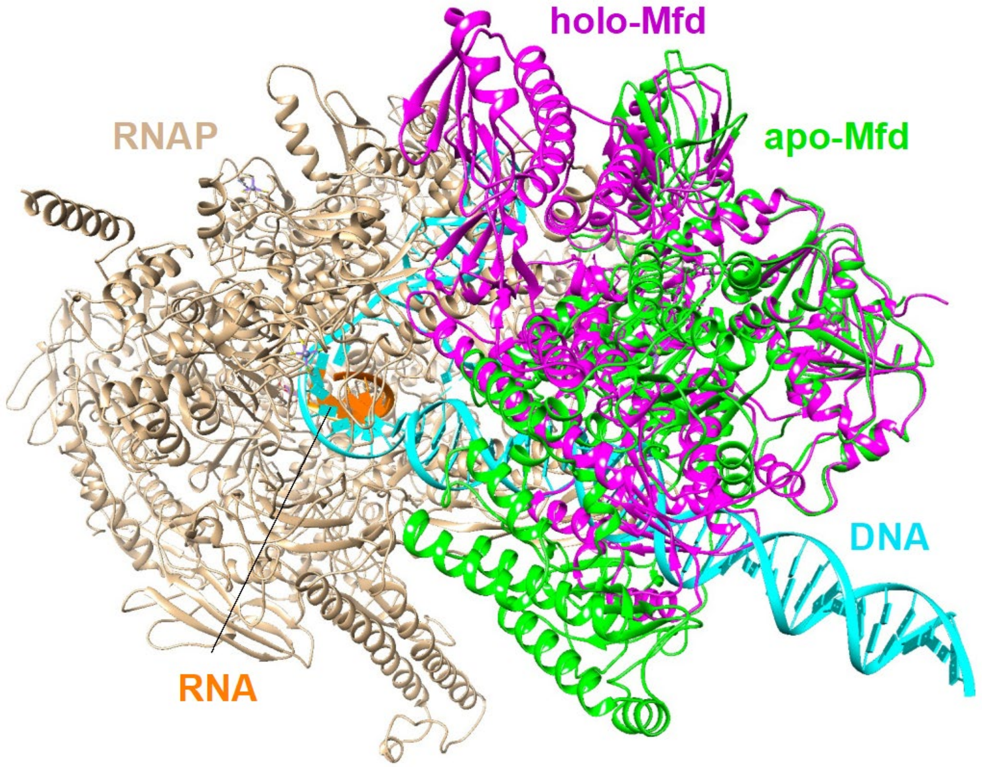

Fig. S5. Superimposition of *E. coli* Mfd in the crystal structure of the enzyme alone (apo form, green; PDB 2eyq) and in the cryo-EM structure of the enzyme bound to RNAP, tan, DNA, cyan, and RNA, orange (holo form, magenta; PDB ID 6x50).

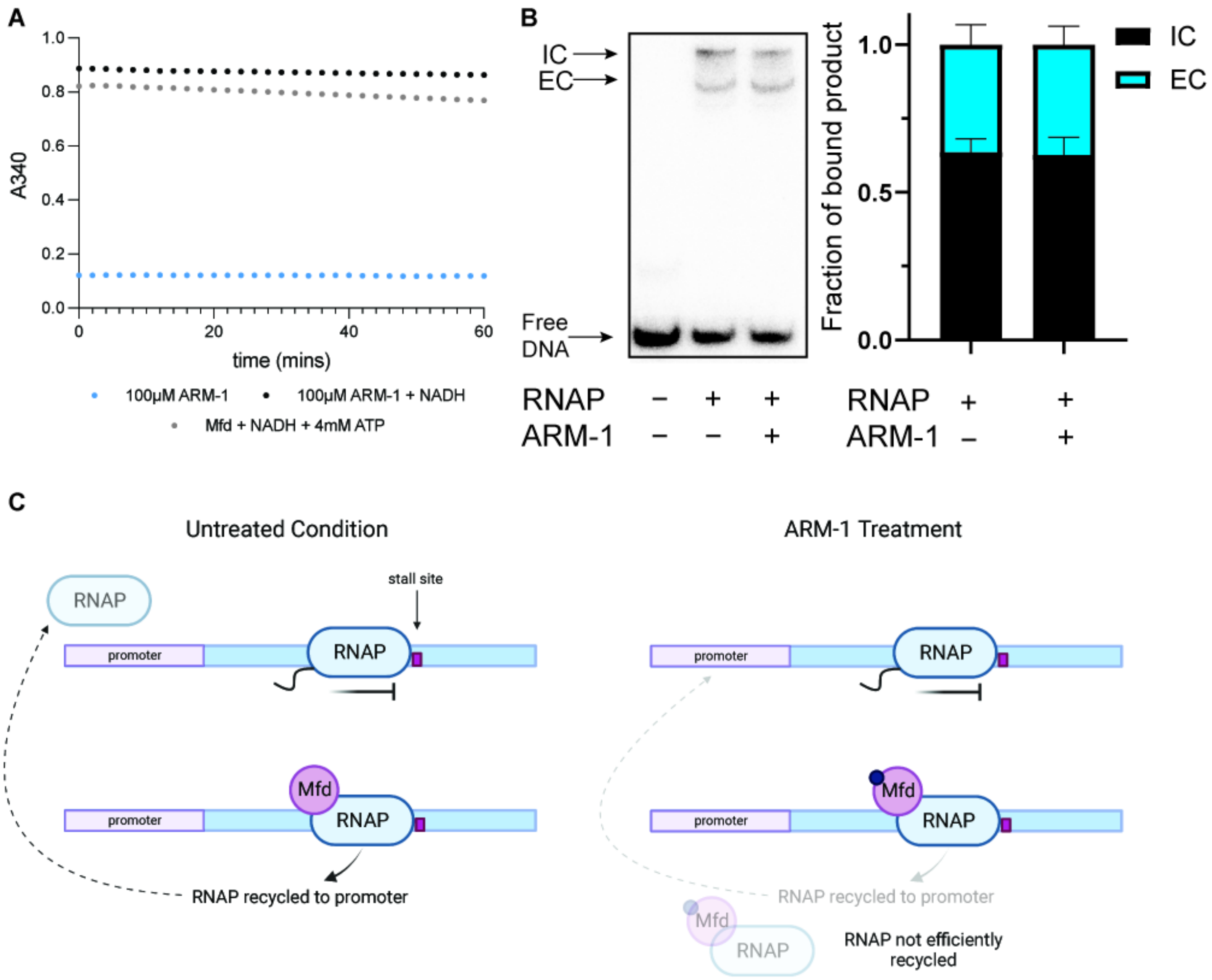

Figure S6: A) NADH-coupled ATPase assay in the absence of Mfd. Absorbance at 340nm by NADH is used to monitor the reaction. B) Proposed model of the ARM-1 effect on Mfd and RNAP. Left: Mfd recognized stalled RNAP and removes it from the DNA, this allows RNAP to be recycled and to bind the promoter again, which increases the population of initiation complexes (ICs) and decreased elongation complexes (ECs) (Fig. 2c, lane 3). Right: when ARM-1 is present Mfd does displace stalled RNAPs (reduced ECs, Fig. 2c, lane 4), but RNAP is unable to re-bind the promoter (no observed increase in IC population, Fig. 2c, lane 4). C) Transcription roadblock assay in the absence of Mfd. Representative gel as well as the average IC and EC intensity of 3 experiments are shown. Error bars represent the SEM.

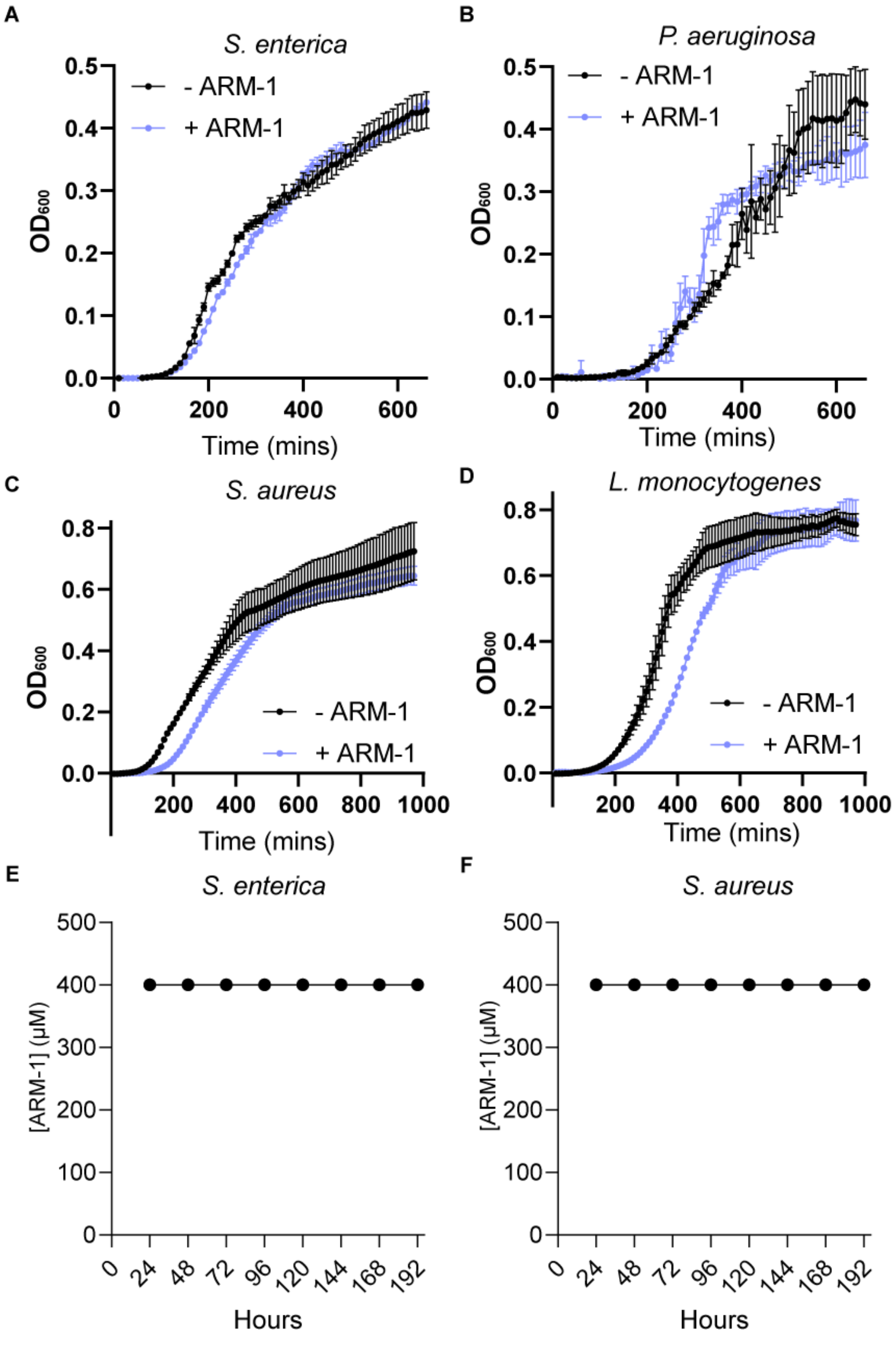

Figure S7. a-d) OD600 measured every 10 mins for the indicated time in a cultures of *S. enterica* (a), *P. aeruginosa* (b), *S. aureus* (c), and *L. monocytogenes* (d), with and without 100 μM ARM-1 in the media. n=4 (*S. enterica*, *P. aeruginosa*) or 8 (*S. aureus, L. monocytogenes*) biological

replicates. e-f) Evolution of indicated species against ARM-1. Median MICs at the indicated timepoints are indicated. timepoints. n=12 biological replicates.

Supplementary Tables

Table S1: Strains

**Strain**	**Source**	**Identifier**
E. coli NM525 Δmfd	Gift from Dr. Deaconescu (Brown University) (1)	
E. coli NM525 Δmfd + pRCB-Nluc + pUC19-Mfd	This study	HM3419
E. coli NM525 Δmfd + pRCB-Nluc + pUC19	This study	HM3664
S. enterica ST19	(2)	HM1996
S. aureus	(3)	HM4318
L. monocytogenes 10403S	Gift from Dr. Woodward (Univ. of Washington)	HM3274
P. aeruginosa CF127	(4)	HM2212

Table S2: Primers

**HM#**	**Sequence**	**Target**
3564	TAAATAAGCTAGCTTTACCTGTTCGGCGC	*S. enterica mfd*
3565	TAAATAACTCGAGTTATGCAATAGCGTTTTCTTC	*S. enterica mfd*
3566	TTATTTACTCGAGTGGGGTGCCTAATGAGT	pUC19
3567	TTATTTAGCTAGCCGGTATTTTCTCCTTACGC	pUC19
3568	GGGGATGGGGAGTAAGCTTACGCCAGAATGCGTT	pNL1
3569	GTCCACGCTAGATCTGAATTCATGGTCTTCACACTCGAAGAT	pNL1
3570	GCTTACTCCCCATCCCC	pRGB-KA4
3571	GAATTCAGATCTAGCGTGGAC	pRGB-KA4
6523	TGTTTTGTCATGGTCGTTTTCC	*S. aureus folA*
6524	AAATGAAATGATAGAAGAAGGAGGATAATT	*S. aureus folA*
6525	TACTACAAAACCAATTCCGTATCG	*S. aureus rpoB* RRDR
6526	TAGCCATAACTGTATTGTTACCAC	*S. aureus rpoB* RRDR
6636	ATTGCGCTTTACGTATAGTGGCG	*S. enterica folA*
6637	TGGCTCAAGAGTAGGCCGGATAAG	*S. enterica folA*
6696	TTTCATCCATTAGCTACTTCTTTAACTTGCTA	*L. monocytogenes rpoB*
6697	TTAGGAGATACATCCATGTAGTCAATACG	*L. monocytogenes rpoB*

Supplemental references.

1. Deaconescu,A.M., Chambers,A.L., Smith,A.J., Nickels,B.E., Hochschild,A., Savery,N.J. and Darst,S.A. (2006) Structural Basis for Bacterial Transcription-Coupled DNA Repair. *Cell*, **124**, 507–520.

2. Hayden,H.S., Matamouros,S., Hager,K.R., Brittnacher,M.J., Rohmer,L., Radey,M.C., Weiss,E.J., Kim,K.B., Jacobs,M.A., Sims-Day,E.H., *et al.* (2016) Genomic Analysis of Salmonella enterica Serovar Typhimurium Characterizes Strain Diversity for Recent U.S. Salmonellosis Cases and Identifies Mutations Linked to Loss of Fitness under Nitrosative and Oxidative Stress. *mBio*, **7**.

3. Carvajal-Garcia,J., Samadpour,A.N., Hernandez Viera,A.J. and Merrikh,H. (2023) Oxidative stress drives mutagenesis through transcription-coupled repair in bacteria. *Proceedings of the National Academy of Sciences*, **120**.

4. Wolfgang,M.C., Kulasekara,B.R., Liang,X., Boyd,D., Wu,K., Yang,Q., Miyada,C.G. and Lory,S. (2003) Conservation of genome content and virulence determinants among clinical and environmental isolates of *Pseudomonas aeruginosa*. *Proceedings of the National Academy of Sciences*, **100**, 8484–8489.
